# Angular Upsampling in Infant Diffusion MRI Using Neighborhood Matching in **x**-**q** Space

**DOI:** 10.3389/fninf.2018.00057

**Published:** 2018-09-07

**Authors:** Geng Chen, Bin Dong, Yong Zhang, Weili Lin, Dinggang Shen, Pew-Thian Yap

**Affiliations:** ^1^Department of Radiology and Biomedical Research Imaging Center (BRIC), University of North Carolina at Chapel Hill, Chapel Hill, NC, United States; ^2^Beijing International Center for Mathematical Research, Peking University, Beijing, China; ^3^Vancouver Research Center, Huawei Technologies Canada, Burnaby, BC, Canada; ^4^Department of Brain and Cognitive Engineering, Korea University, Seoul, South Korea

**Keywords:** diffusion MRI, upsampling, non-local means, neighborhood matching, regularization

## Abstract

Diffusion MRI requires sufficient coverage of the diffusion wavevector space, also known as the *q*-space, to adequately capture the pattern of water diffusion in various directions and scales. As a result, the acquisition time can be prohibitive for individuals who are unable to stay still in the scanner for an extensive period of time, such as infants. To address this problem, in this paper we harness non-local self-similar information in the *x*-*q* space of diffusion MRI data for *q*-space upsampling. Specifically, we first perform neighborhood matching to establish the relationships of signals in *x*-*q* space. The signal relationships are then used to regularize an ill-posed inverse problem related to the estimation of high angular resolution diffusion MRI data from its low-resolution counterpart. Our framework allows information from curved white matter structures to be used for effective regularization of the otherwise ill-posed problem. Extensive evaluations using synthetic and infant diffusion MRI data demonstrate the effectiveness of our method. Compared with the widely adopted interpolation methods using spherical radial basis functions and spherical harmonics, our method is able to produce high angular resolution diffusion MRI data with greater quality, both qualitatively and quantitatively.

## 1. Introduction

Infant brain development involves complex cerebral growth and maturation with the white matter (WM) undergoing rapid myelination and synaptogenesis (Qiu et al., 2015). Diffusion MRI (DMRI) has been widely employed to study this developmental process *in vivo* (Yap et al., 2011; Huang et al., 2013; Dubois et al., 2014; Qiu et al., 2015). For instance, using diffusion tensor imaging (DTI), researchers have observed an increase in the fractional anisotropy (FA) during the first few years of life (Dubois et al., 2014; Qiu et al., 2015), implying more restriction on water movement owing to the ensheathment of oligodendrocytes around the axons. Mean diffusivity (Dubois et al., 2014; Qiu et al., 2015) and structural connectivity (Yap et al., 2011; Huang et al., 2013) have also been used to study early brain development.

Existing brain development studies mainly rely on DTI. However, DTI utilizes a simple but insufficient tensor model to characterize local fiber configurations. More sophisticated diffusion models are needed to better characterize realistic configurations, such as crossing, fanning, and bending, and to tease out inter- and extra-cellular compartments (Ning et al., 2015; Yap et al., 2016b; Ye et al., 2016). However, unlike the tensor model that only requires 6 diffusion-weighted (DW) images and one non-DW image, these advanced models requires high angular resolution (HAR) DMRI data. The angular resolution of DMRI data is determined by the number of gradients used in data acquisition. Each gradient corresponds to a point in *q*-space. A larger number of DW images allows the use of advanced diffusion models but prolong the acquisition time, which is prohibitive in clinical settings.

In practice, the window of opportunity for imaging infants is short. To put this in perspective, in the Human Connectome Project (HCP) (Van Essen et al., 2012) each individual was allotted a DMRI scan time of about an hour. However, in the Baby Connectome Project (BCP) (Fallik, 2016; Cao et al., 2017; Howell et al., 2018), the tolerable scan time is well below 15 min. Infants are typically scanned without sedation while they are asleep. The scanning may also need to be terminated prematurely if the infant is awakened by the loud acoustic noise and sudden vibrations caused by the rapid switching of gradient amplitude and polarity (McJury and Shellock, 2000; Hutter et al., 2017). The short acquisition time precludes a denser coverage of *q*-space, limiting studies to simpler models such as the diffusion tensor.

To increase the angular resolution without inducing additional acquisition time, post-acquisition angular upsampling methods have been proposed. Tuch (2004) proposed to interpolate *q*-space using spherical radial basis functions (SRBFs). This method interpolates one point in *q*-space by weighted averaging of angularly neighboring *q*-space measurements. An alternative method is interpolation using spherical harmonics (SHs) (Descoteaux et al., 2007). For each voxel, this method first decomposes the DMRI measurements into an SH coefficient vectors. DMRI measurements are then reconstructed with the coefficients and the corresponding SH basis. Despite the promising performance of these methods, one major limitation is that only *q*-space information is considered during interpolation, and valuable *x*-space information is overlooked.

To overcome this limitation, in this paper, we propose to harness joint *x*-*q* space information for angular upsampling of DMRI data. For this purpose, we first establish signal correspondences in *x*-*q* space using a robust neighborhood matching technique described in Chen et al. (2016a, 2017a). We then recover the HAR DMRI data in a regularization framework based on the signal correspondences. Angular upsampling is achieved by using the signal relationships in the joint *x*-*q* space. Extensive experiments on synthetic and infant DMRI data demonstrate that our method is able to recover HAR DMRI data with a remarkably improved quality.

Part of this work has been reported in our recent conference paper (Chen et al., 2017b). Herein, we present (1) New insights into the application of our method to infant DMRI data; (2) More detailed descriptions of the proposed method; (3) More complete mathematical derivation details; (4) Comparison with the state-of-the-art method, SH interpolation; (5) New experimental results on synthetic data and infant data acquired at different time points. None of these is part of the conference publication.

The rest of the paper is organized as follows. In Section 2, we give a detailed description of the proposed method. In Section 3, we demonstrate the effectiveness of the method with both synthetic and real infant DMRI data. In Section 4, we provide further discussion on this work. Finally, in Section 5, we conclude this work.

## 2. Methods

Each signal in the *x*-*q* space is associated with a voxel location in the *x*-space and a set of diffusion gradient direction and strength in the *q*-space. The signals are typically collected via a set of DW images, each corresponding to a point in *q*-space. In this section, we will first detail in how to establish the signal relationships in *x*-*q* space and then clarify how to utilize these relationships to regularize the ill-posed inverse problem associated with angular upsampling of DMRI data.

### 2.1. Signal correspondences in *x*-*q* space

**Figure 1:**
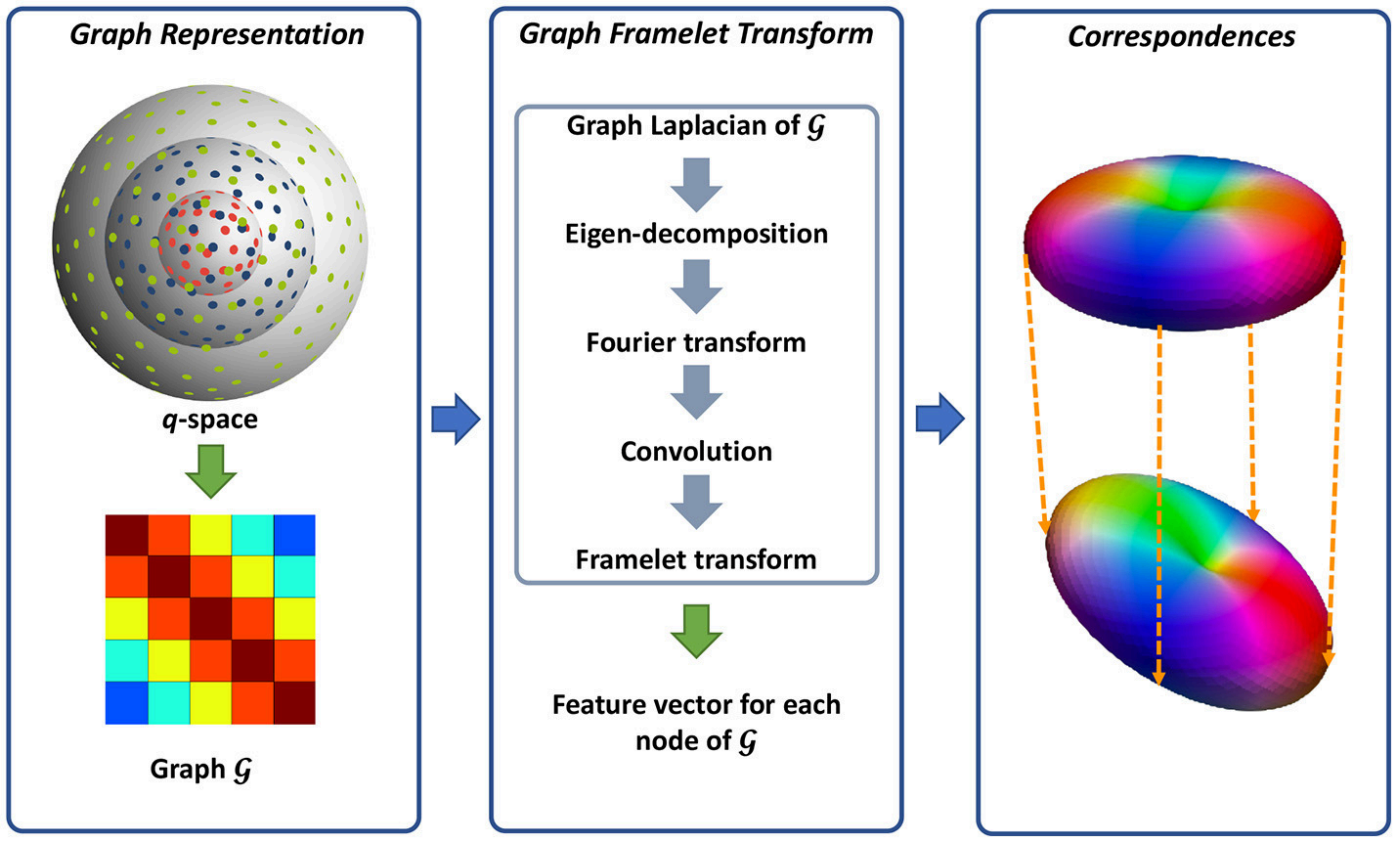
Overview. **(Left)** Representing the *q*-space sampling domain using a graph with affinity matrix determined by kernels for diffusion gradient directions and strengths. **(Middle)** Feature computation using GFTs. **(Right)** Neighborhood matching.

We utilize a neighborhood matching technique to determine signal correspondences in x-q space. Neighborhood matching techniques have a wide range of applications in medical image analysis, including statistical group comparison (Chen et al., 2015), atlas building (Yang et al., 2017; Saghafi et al., 2017; Kim et al., 2017), fiber orientation estimation (Chen et al., 2016b), and denoising (Chen et al., 2016a,c). Instead of using the conventional methods designed mainly for x-space (Buades et al., 2005; Chen et al., 2016c), we employ the method described in Chen et al. (2016a, 2017a) for accurately establishing signal correspondences in x-q space. The signal relationships in x-q space is quantified using similarity weights (Chen et al., 2016a, 2017a). A large weight is assigned to two matching signals, while the weight of two mismatching signals is low. To compute the similarity weight, robust rotation-invariant features are first computed for each node in *x*-*q* space. As in Chen et al. (2016a, 2017a), we represent the *q*-space using a graph and employ a technique, named graph framelet transforms (GFTs), for the feature computation. The resulting features are then employed to compute the similarity weights, essentially establishing the signal correspondence in *x*-*q* space. Figure 1 illustrates an overview of this method. We flesh out these steps in the following.

#### 2.1.1. Graph representation for *q*-space

As illustrated in Figure 1, we use a graph G to represent the *q*-space. For this purpose, we compute the adjacency weight between each two nodes in *q*-space using two kernels for diffusion gradient directions and strengths. In this way, the geometric relationships in *q*-space are encoded in the graph G where a large edge weight indicates two nodes sharing similar gradient directions and diffusion weightings. Since *q*-space is now represented as a graph, we can view the signals at each voxel as a function *f* defined on the graph.

#### 2.1.2. Feature computation

We then compute the multi-scale features using GFTs. The key idea of GFT is to slice the frequency spectrum of *f* in a multi-scale fashion by using a set of masks. This involves how to perform convolutions on a graph. As clarified in Dong (2017), the eigenvectors and eigenvalues of graph Laplacian are the Fourier spectrum and basis of one graph. Therefore, the convolution can be defined in the transform domain by slicing the Fourier spectrum using a mask. In this way, the graph framelet analysis transform **W** up to level *L* and mask *R* can be defined as

(1) $$\begin{array}{r} \text{α}:=\text{W}f:=\left\{\alpha_{l,r}:=W_{l,r}f:\left(l,r\right)\in B_{L,R}\right\}, \end{array}$$

where $B_{L,R}:=\left\{\left(1,1\right),\left(1,2\right),\ldots ,\left(1,R\right),\left(2,1\right),\ldots ,\left(L,R\right)\right\}\cup \left\{\left(L,0\right)\right\}$ and *W*_*l, r*_ is a matrix representing the GFT operator associated with *l* and *r*.

We then have the feature vector for graph node *k* as $\phi \left[k\right]:=\left\{\alpha_{l,r}\left[k\right]:\left(l,r\right)\in B_{L,R}\right\}$. GFT has three major advantages: (1) Due to the multi-scale nature, it provides rich characterizations of the signal information; (2) As proven in Yap et al. (2016a); Dong (2017), the GFT feature is rotation-invariant, allowing the neighborhood matching in the curved domain. (3) As shown in Equation (1), GFT is linear and thus computationally efficient.

#### 2.1.3. Neighborhood matching in *x*-*q* space

The resulting GFT features are then employed for neighborhood matching. We define the similarity weight as a Gaussian function of GFT feature distance, i.e.,

(2) $$w[k;l]=G_{\text{GFT}}(||\phi [k]-\phi [l]||).\%G({\sqrt{b}}_{k}-{\sqrt{b}}_{l}),$$

For the *x-q* space neighborhood matching in DMRI data, we extend Equation (2) with the consideration of spatial location and diffusion-weighting variations. The new form of Equation (2) is

(3) $$w[i,k;j,l]=G_{\text{GFT}}(||\phi_{i}[k]-\phi_{j}[l]||)G_{\text{b}}({\sqrt{b}}_{k}-{\sqrt{b}}_{l}),$$

where *i* and *j* are the indices of two spatial locations; *b*_*k*_ is the *b*-value for the gradient with index *k*. Through neighborhood matching, similarity weights are assign to paired nodes in *x*-*q* space, ultimately establishing dense node-to-node correspondences.

### 2.2. Angular upsampling

We use the *x*-*q* space data relationships determined in the previous section to guide data upsampling in the *q*-space. In general, recovering the high angular resolution (HAR) data from the low angular resolution (LAR) data is an ill-posed inverse problem. Solution can however be feasible by imposing structure via harnessing prior information to reduce the dimensionality of the problem. Our approach uses the signal correlation in the *x*-*q* space to help reduce the complexity of the problem by imposing that the reconstructed signal should be smooth in a non-local sense. That is, neighboring points in the product space of the signal space and the *x*-*q* space should be reconstructed using similar values. Unlike the commonly used *x*-space regularization (Coupé et al., 2013), which takes into account spatial correlation, *x*-*q* space regularization allows correlation across DW images collected using different gradient directions and strengths to be considered. This is fitting when considering the fact that WM structures might be curved and hence causing rapid changes within a DW image.

We formulate the angular upsampling problem in a regularization framework and define our objective function as

(4) $$\begin{array}{l} \epsilon^{2}(x)=\frac{\lambda }{2}\underset{\text{Data Fidelity Term}}{\underset{︸}{\|Ax-y{\|}_{2}^{2}}}\\ \;+\underset{\text{Regularization Term}}{\underset{︸}{\frac{1}{4}\sum_{(i,k)\in \Omega }\sum_{\left(j,l)\in V(i,k\right)}w[i,k;j,l]\|R_{i,k}x-R_{j,l}x{\|}_{2}^{2}}}, \end{array}$$

where **x** and **y** are two vectors representing the HAR and LAR data, respectively; **A** is a *q*-space downsampling operator; **R**_*i, k*_ is an operator that extracts the diffusion signal associated with index (*i, k*). The penalty function consists of a data fidelity term and a regularization term based on *x*-*q* space neighborhood matching. The data fidelity term well preserves the information of original LAR data, while the regularization term encourages that each signal to be represented by its matching signals, essentially establishing the non-local smoothness (Protter et al., 2009).

### 2.3. Optimization

To minimize (4), we compute the derivative and equate it to zero:

(5) $$\begin{array}{l} 0=\frac{d\epsilon^{2}(x)}{dx}\\ \;=\lambda A^{⊤}(Ax-y)+\frac{1}{2}\sum_{(i,k)\in \Omega }\sum_{\left(j,l)\in V(i,k\right)}w[i,k;j,l]{\left(R_{i,k}-R_{j,l}\right)}^{⊤}\\ \;(R_{i,k}-R_{j,l})x\\ =\lambda A^{⊤}(Ax-y)+\frac{1}{2}\sum_{(i,k)\in \Omega }\sum_{\left(j,l)\in V(i,k\right)}w[i,k;j,l]R_{i,k}^{⊤}R_{i,k}x\\ \;-\frac{1}{2}\sum_{(i,k)\in \Omega }\sum_{\left(j,l)\in V(i,k\right)}w[i,k;j,l]R_{i,k}^{⊤}R_{j,l}x\\ \;-\frac{1}{2}\sum_{(i,k)\in \Omega }\sum_{\left(j,l)\in V(i,k\right)}w[i,k;j,l]R_{j,l}^{⊤}R_{i,k}x\\ \;+\frac{1}{2}\sum_{(i,k)\in \Omega }\sum_{\left(j,l)\in V(i,k\right)}w[i,k;j,l]R_{j,l}^{⊤}R_{j,l}x. \end{array}$$

Based on the facts that the neighborhood is symmetric (i.e., if (j,l)∈V(i,k), then (i,k)∈V(j,l)) and the weights are symmetric (i.e., *w*[*i, k*; *j, l*] = *w*[*j, l*; *i, k*]) (Protter et al., 2009), we have

(6) $$\begin{array}{l} \sum_{(i,k)\in \Omega }\sum_{\left(j,l)\in V(i,k\right)}w[i,k;j,l]R_{i,k}^{⊤}R_{i,k}x\\ \;=\sum_{(i,k)\in \Omega }\sum_{\left(j,l)\in V(i,k\right)}w[i,k;j,l]R_{j,l}^{⊤}R_{j,l}x\\ \sum_{(i,k)\in \Omega }\sum_{\left(j,l)\in V(i,k\right)}w[i,k;j,l]R_{j,l}^{⊤}R_{i,k}x\\ \;=\sum_{(i,k)\in \Omega }\sum_{\left(j,l)\in V(i,k\right)}w[i,k;j,l]R_{i,k}^{⊤}R_{j,l}x. \end{array}$$

Equation (5) can be simplified using (6), giving

(7) $$\begin{array}{l} 0=\lambda A^{⊤}(Ax-y)+\sum_{(i,k)\in \Omega }\sum_{\left(j,l)\in V(i,k\right)}w[i,k;j,l]R_{i,k}^{⊤}R_{i,k}x\\ \;-\sum_{(i,k)\in \Omega }\sum_{\left(j,l)\in V(i,k\right)}w[i,k;j,l]R_{i,k}^{⊤}R_{j,l}x. \end{array}$$

Equation (7) can be solved directly but involves the inversion of a very large matrix, therefore we choose instead to use fixed-point iteration to solve the problem, as suggested in Protter et al. (2009). If we let **x**^*n*^ be the solution at iteration *n*, the following can be proven to be convergent (Protter et al., 2009):

(8) $$\begin{array}{l} 0=\lambda A^{⊤}(Ax^{n}-y)+\sum_{(i,k)\in \Omega }\sum_{\left(j,l)\in V(i,k\right)}w[i,k;j,l]R_{i,k}^{⊤}R_{i,k}x^{n}\\ \;-\sum_{(i,k)\in \Omega }\sum_{\left(j,l)\in V(i,k\right)}w[i,k;j,l]R_{i,k}^{⊤}R_{j,l}x^{n-1}. \end{array}$$

The solution **x** can be obtained iteratively using

(9) $$\begin{array}{l} x^{n}={\left(\lambda A^{⊤}A+\sum_{(i,k)\in \Omega }\sum_{\left(j,l)\in V(i,k\right)}w[i,k;j,l]R_{i,k}^{⊤}R_{i,k}\right)}^{-1}\\ \;\times \left(\lambda A^{⊤}y+\sum_{(i,k)\in \Omega }\sum_{\left(j,l)\in V(i,k\right)}w[i,k;j,l]R_{i,k}^{⊤}R_{j,l}x^{n-1}\right). \end{array}$$

Note that **A**^⊤^**A** is an identical matrix and $\underset{\left(i,k\right)\in \Omega }{\sum }\underset{\left(j,l\right)\in V\left(i,k\right)}{\sum }w\left[i,k;j,l\right]{\text{R}}_{i,k}^{⊤}{\text{R}}_{i,k}$ is a diagonal matrix, therefore the matrix inversion in (9) can be done effectively.

### 2.4. Implementation issues

#### 2.4.1. Initialization

The data are transformed so that the noise is Gaussian distributed as described in Koay et al. (2009). The algorithm is then initialized using an upsampled version of **y**, which is obtained via interpolation using SHs.

#### 2.4.2. Neighborhood matching

Neighborhood matching is performed based on the upsampled version of **y**. The resulting weights remain unchanged until a solution **x** is obtained. In principle, we can use **x** to re-estimate the weights and rerun the algorithm to obtain a refined solution. However, our experimental results indicate that the benefit of doing so is minimal. Therefore, we will only show results without weight re-estimation.

#### 2.4.3. Stopping criterion

We stop the algorithm when the mean absolute difference (MAD) between the outcomes of two iterations, i.e., **x**^*n*−1^ and **x**^*n*^, is less than a constant tol. We define tol = *β*σ_G_, where σ_G_ is the standard derivation of the Gaussian noise and *β* is a constant.

## 3. Experiments

The proposed angular upsampling method was evaluated using both synthetic and real data. Through grid search, we found that λ = 100 and *β* = 10^−3^ give the best results. We compared the proposed method with two baselines, SRBF interpolation and SH interpolation.

### 3.1. Datasets

#### 3.1.1. Synthetic data

For quantitative evaluation, we generated a set of synthetic data using phantomαs (Caruyer et al., 2014) and the fiber geometric model of ISBI 2013 HARDI challenge^1^. We utilized 321 gradient directions, uniformly distributed on the surface of a sphere, to simulate the HAR DMRI data. The number of gradient directions was reduced to 81 for the simulation of corresponding LAR counterpart. In the data simulation, we used three *b*-values, including 1, 000, 2, 000, 3, 000s/mm^2^. After obtaining the noise-free LAR data, we added four levels (SNR = 15, 20, 25, 30) of 32-channel noncentral chi (nc-χ) noise to the data to simulate noise disturbances. The noise-free HAR DMRI data was used as the ground truth for quantitative evaluations.

#### 3.1.2. Real data

DMRI data were acquired for three infants at three different time points: 0 month, 6 months, and 12 months. All enrolled subjects had written informed consent provided by parent/guardian. The experimental protocols were approved by the Institutional Review Board of the University of North Carolina (UNC) School of Medicine. The study was carried out in accordance with the approved guidelines. All the data are acquired using a Siemens 3T Magnetom Prisma MR scanner and a standard imaging protocol: 140 × 140 imaging matrix, 1.5 × 1.5 × 1.5 mm^3^ resolution, TE = 88ms, TR = 2, 365ms, 32-channel receiver coil, *b* = 700, 1500, 3000s/mm^2^, and 144 non-collinear gradient directions. We uniformly selected 72 gradient directions to generate the LAR data for evaluation.

### 3.2. Evaluation methods

Quantitative and qualitative evaluations were performed as described in the following:

1. **RMSE maps:** We computed voxel-wise RMSE value between two sets of DMRI datasets to measure their similarity locally.
2. **FA images:** We computed the FA images using the iterative weighted tensor fitting method presented in Salvador et al. (2005).
3. **Absolute difference (AD) maps:** We compute the AD map between one FA image and the ground truth FA image to evaluate the performance of the algorithm locally.
4. **Mean normalized absolute difference (MNAD):** We computed MNAD value by 1) computing AD map, 2) normalizing the AD map using the ground truth FA image voxel-wisely, and 3) computing the mean value of the normalized map within the brain region.
5. **Peak signal-to-noise ratio (PSNR):** PSNR is used for quantitative evaluation of FA images, and is defined as
  (10) $$\text{PSNR}=20\log_{10}\frac{\text{MAX}}{\text{RMSE}},$$
  where MAX is the maximum FA value, which is 1 in our case.
6. **Fiber ODFs:** We compute fiber orientation distribution functions (ODFs) using the method presented in Yap et al. (2016b), which caters to multiple tissue types using multi-shell data.

### 3.3. Results

#### 3.3.1. Quantitative comparison – synthetic data

**Figure 2:**
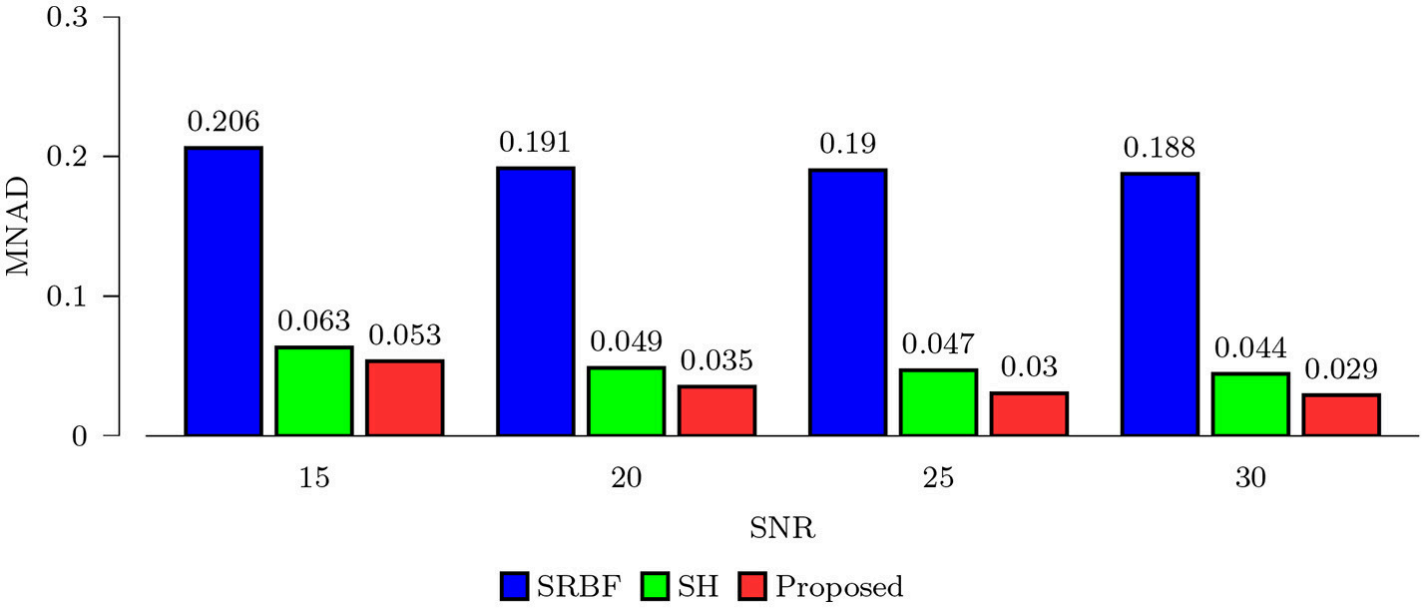
MNAD Comparison – Synthetic Data. Quantitative evaluation using synthetic data via MNAD of FA images.

**Figure 3:**
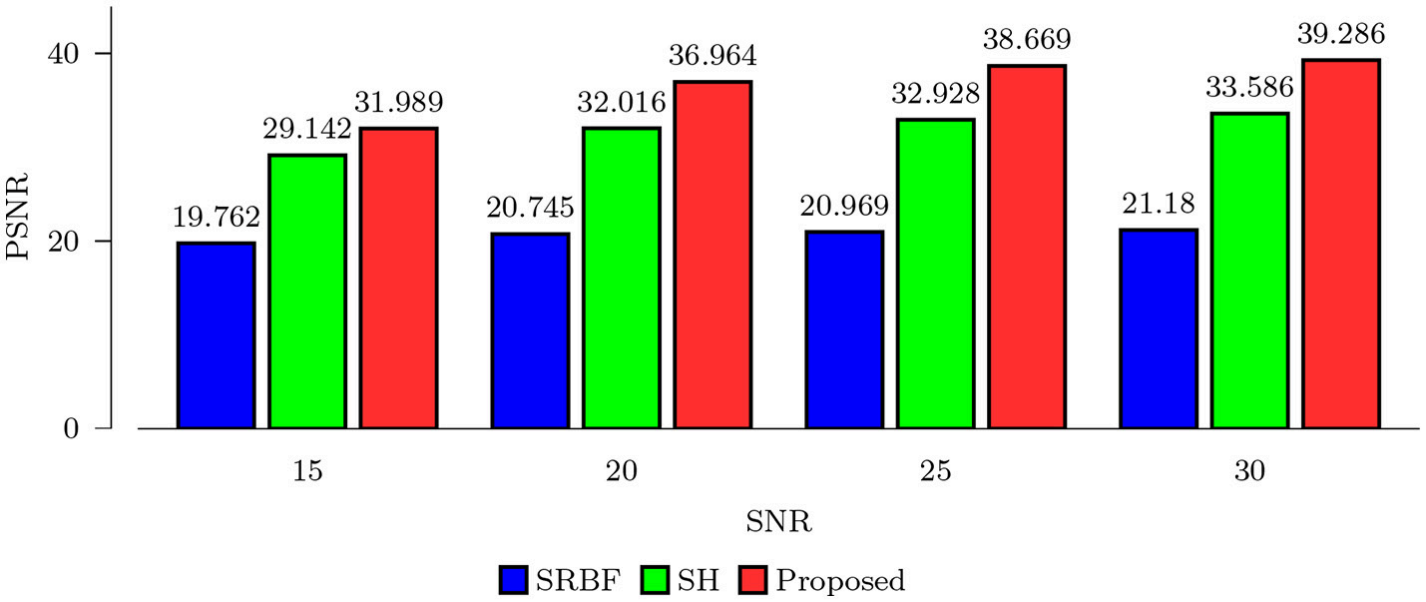
PSNR Comparison – Synthetic Data. Quantitative evaluation using synthetic data via PSNR of FA images.

Using the FA image of noise-free HAR data as ground truth, we evaluated the quality of the upsampled data using MNAD and PSNR. The results, shown in Figures 2 and 3, indicate that the proposed method outperforms SRBF interpolation and SH interpolation for all noise levels. The largest improvement over the second best method, SH interpolation, is 0.017 in term of MNAD when SNR = 25. The corresponding PSNR improvement is 5.74 dB.

#### 3.3.2. Dw images – synthetic data

**Figure 4:**
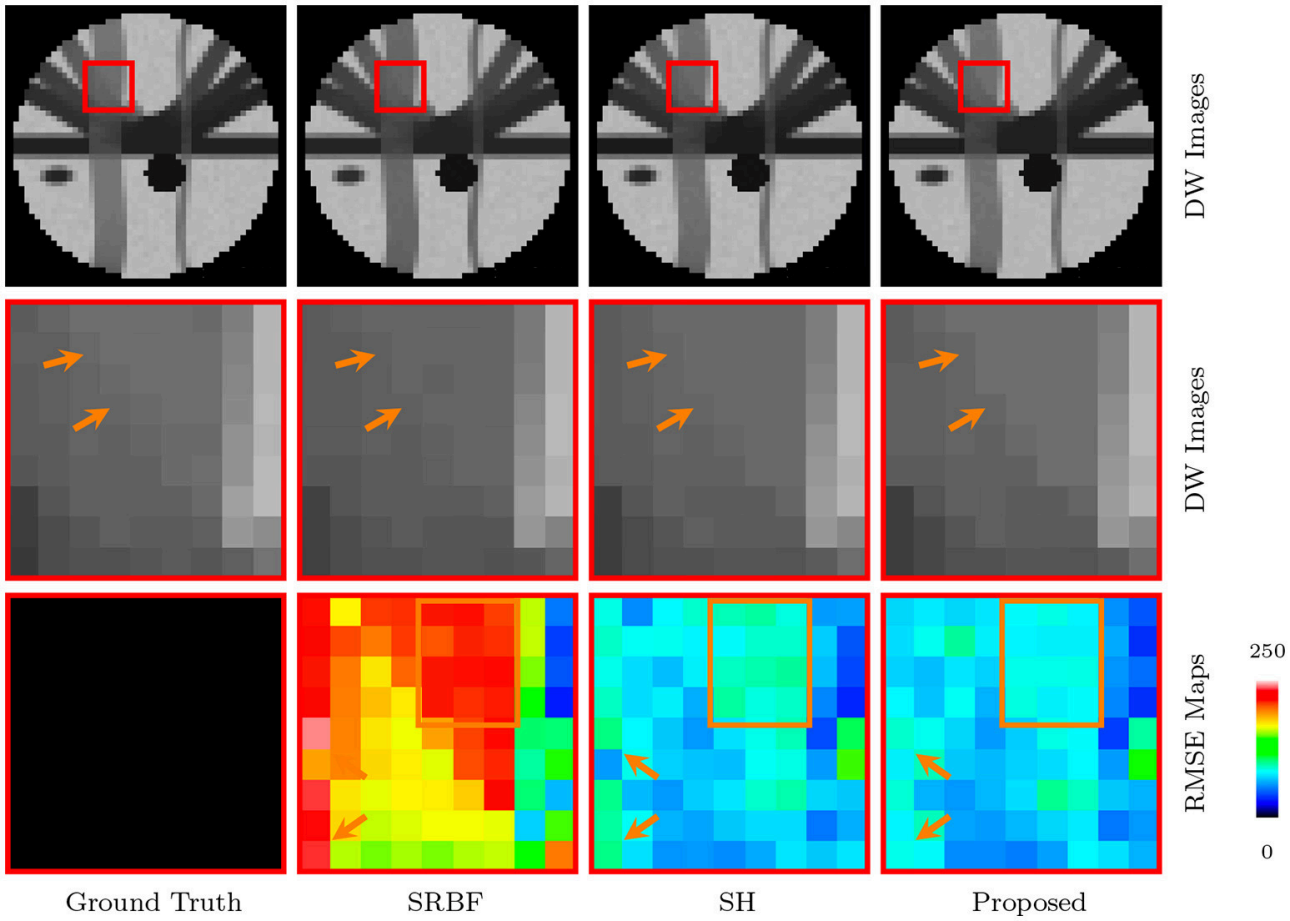
DW Images and RMSE Maps – Synthetic Data. Comparison of upsampling results for *b* = 1, 000s/mm^2^ and nc-χ noise (SNR = 30).

The full views and close-up views of DW images, shown in the top two rows of Figure 4, indicate that the proposed method results in better structural contrast. The close-up views of RMSE maps, shown in the bottom row of Figure 4, indicate that our method gives lower RMSE than SRBF interpolation and SH interpolation, which demonstrates that the upsampled data given by our method is closer to ground truth.

#### 3.3.3. Fa images – synthetic data

**Figure 5:**
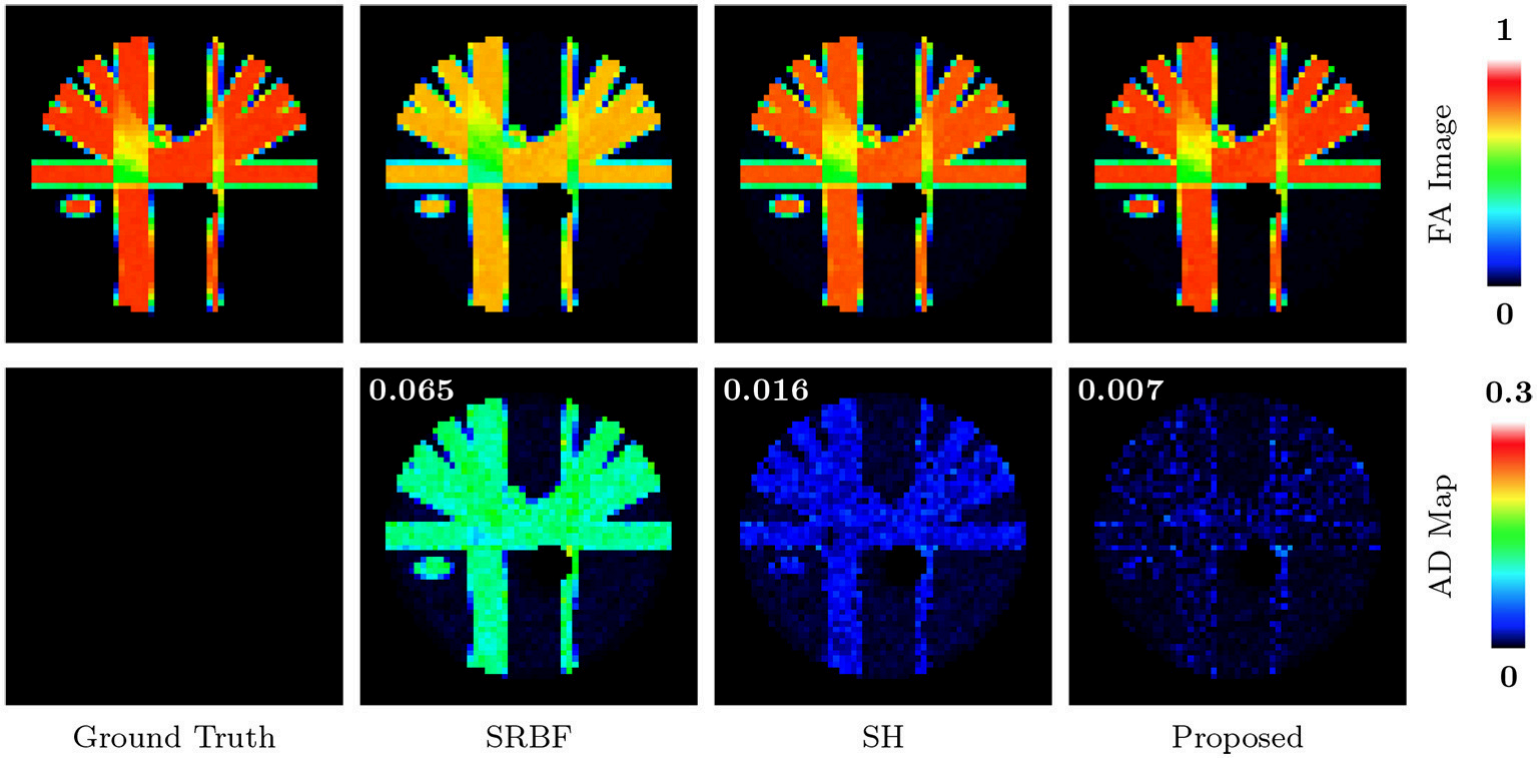
FA Images and AD Maps – Synthetic Data. Evaluation of accuracy in terms of FA using synthetic dataset with nc-χ noise (SNR = 30). The color of FA images represents the FA value, e.g., warmer color means a larger FA value. MAD values are shown at the top left corners.

The top row of Figure 5 shows the FA images given by ground truth data and upsampled data. We use warm colors to represent large FA values for a better visualization. Compared with SRBF interpolation and SH interpolation, our method produces an FA image closer to the ground truth. This observation is further confirmed by the AD maps of the FA images, shown in the bottom row of Figure 5. Our method yields the lowest MAD value, indicating the best performance.

#### 3.3.4. Fiber odfs – synthetic data

**Figure 6:**
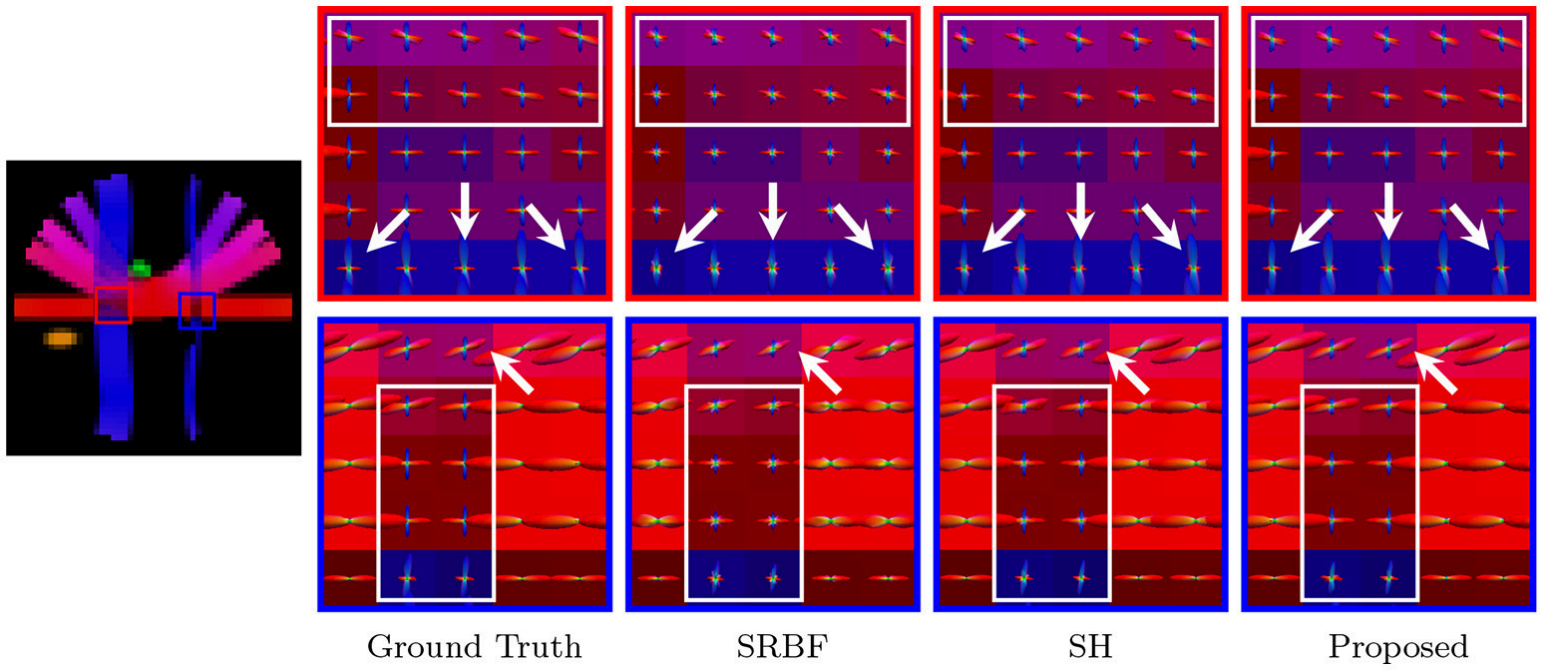
Fiber ODFs – Synthetic Data. Fiber ODF comparison using synthetic data with nc-χ noise (SNR = 30).

Accurate ODF estimation relies on sufficient angular samples. The ODFs, shown in Figure 6, indicate that our method gives clean and coherent ODFs that are close to the ground truth. In contrast, spurious peaks are introduced by SRBF interpolation and SH interpolation.

#### 3.3.5. Diffusion signal profiles – synthetic data

**Figure 7:**
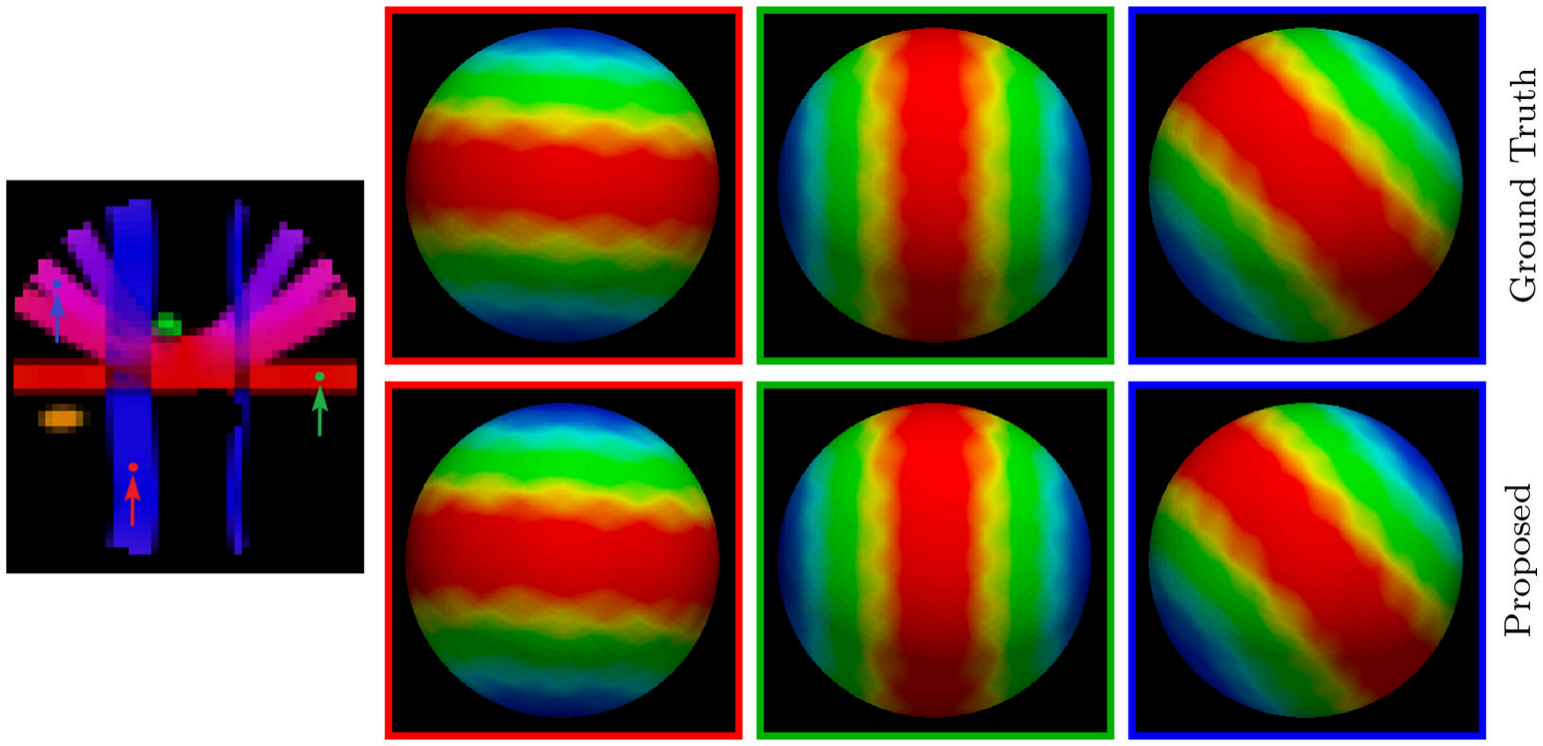
Diffusion Signal Profiles – Synthetic Data. The diffusion signals are rendered on a sphere for visual comparison. The colored FA image is shown on the far left for reference.

For a more direct visualization of the upsampled data, we rendered the signal values on a sphere. The results, shown in Figure 7, indicate that our method gives values that are close to the ground truth.

#### 3.3.6. Mnad comparison – real data

**Figure 8:**
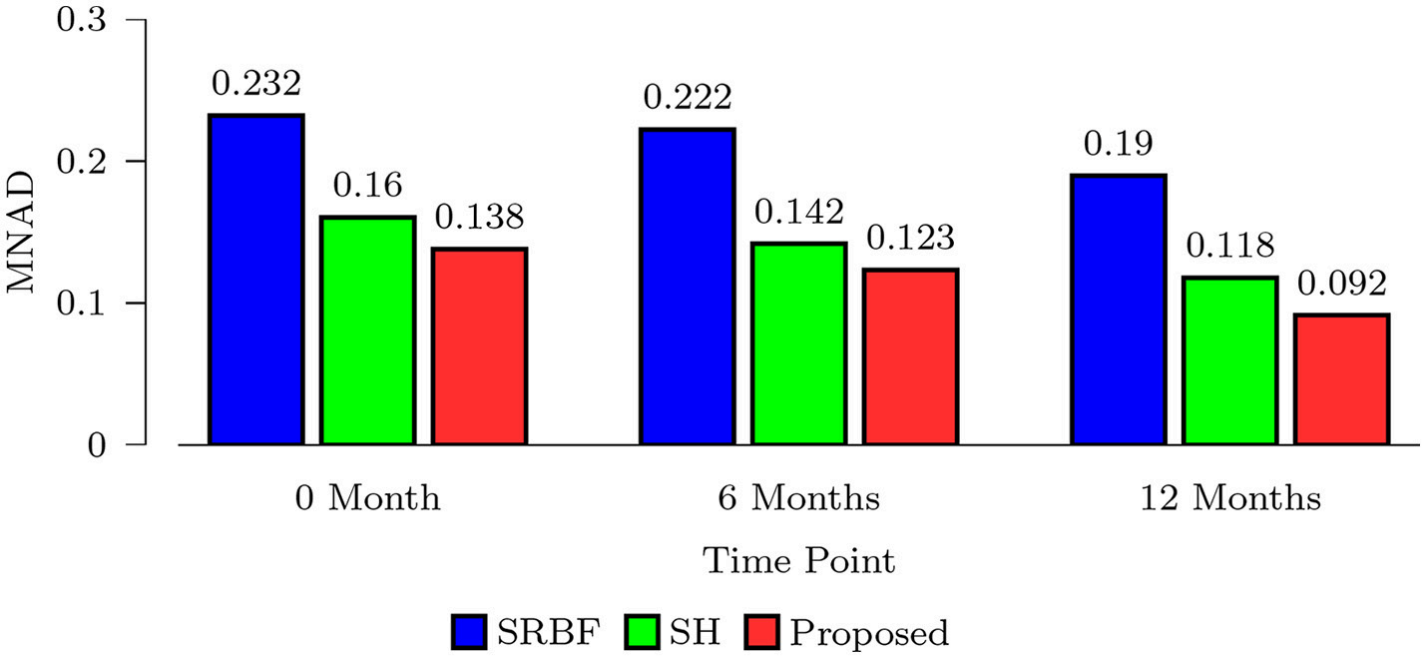
MNAD Comparison – Real Data. Quantitative evaluation using infant data via MNAD of FA images.

We also computed MNAD values for the quantitative evaluation of real data experimental results. Figure 8 shows the MNAD between the FA images given by the upsampled data and the original HAR data. For all time points, our method outperforms SRBF interpolation and SH interpolation, with a largest MNAD reduction of 0.026 over the second best method at 12-months time point.

#### 3.3.7. Dw images – real data

**Figure 9:**
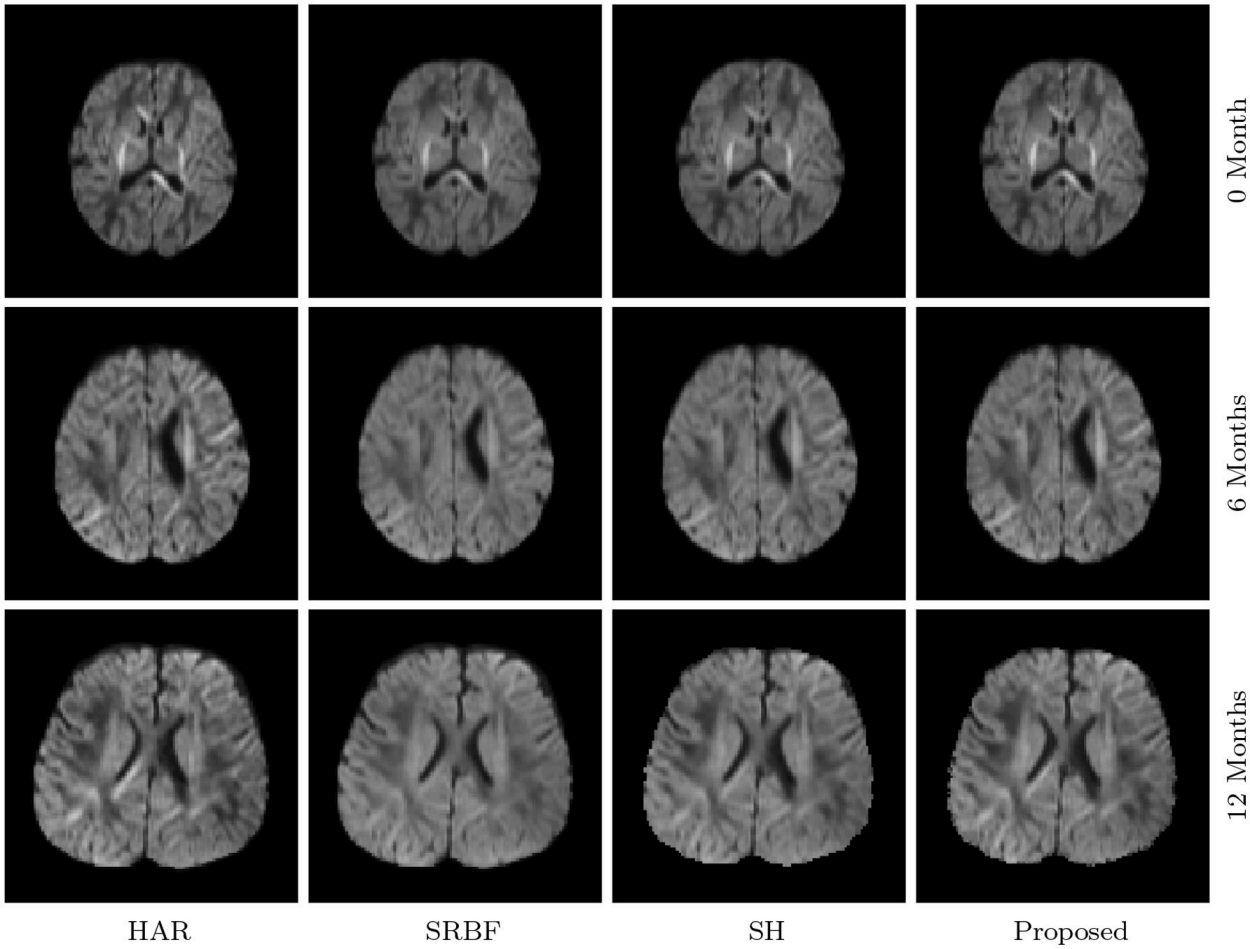
DW Images – Real Data. Comparison of DW images with *b* = 1, 500s/mm^2^.

**Figure 10:**
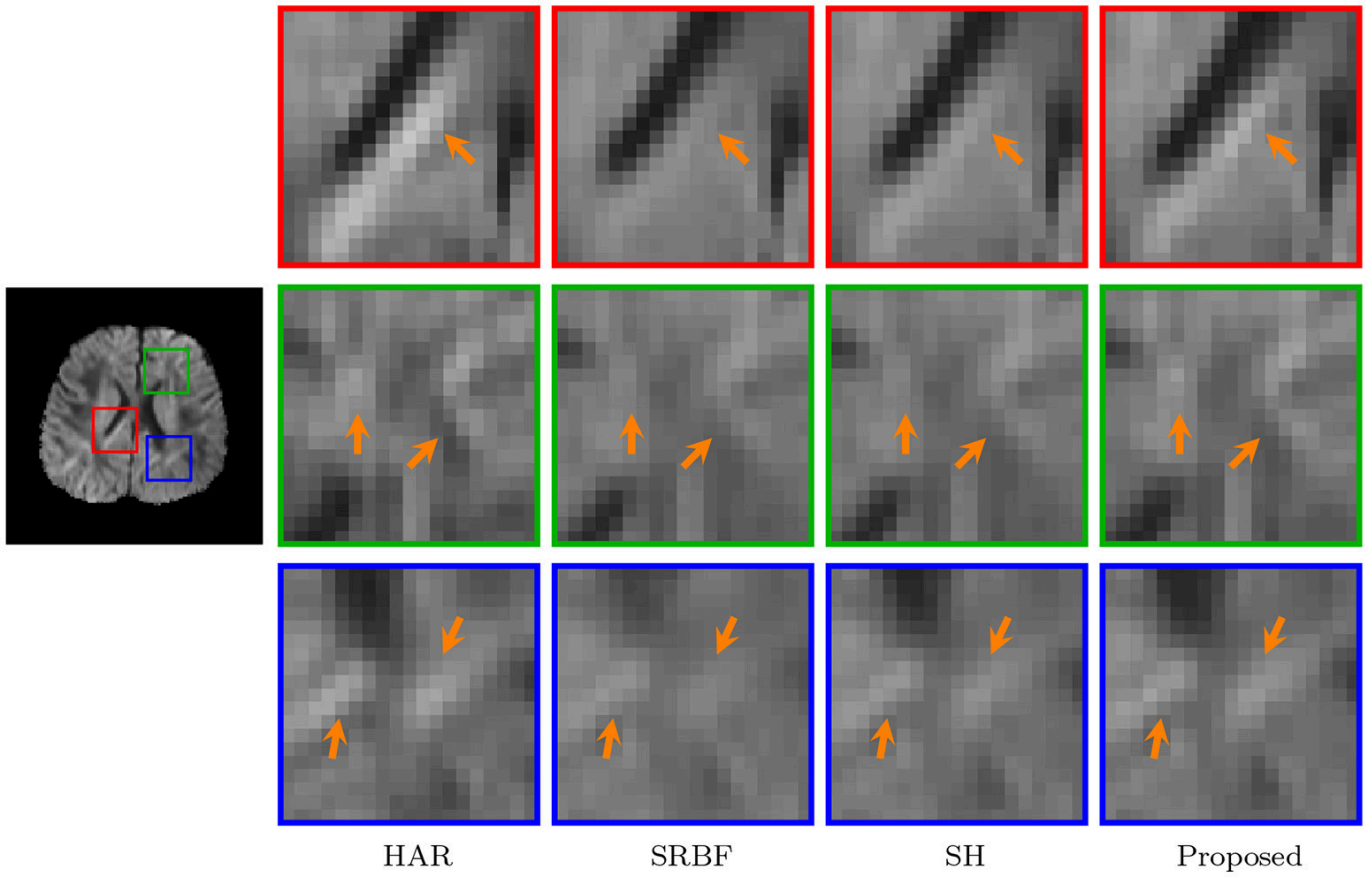
Close-Up Views of DW Images – Real Data. Regional close-up views of the DW images of a 12-month infant subject.

The observations from Figures 9 and 10 for real data are consistent with that in Figure 4. The DW image given by our method shows more subtle structural details and is closer to the one in the original HAR data.

#### 3.3.8. Fa images – real data

**Figure 11:**
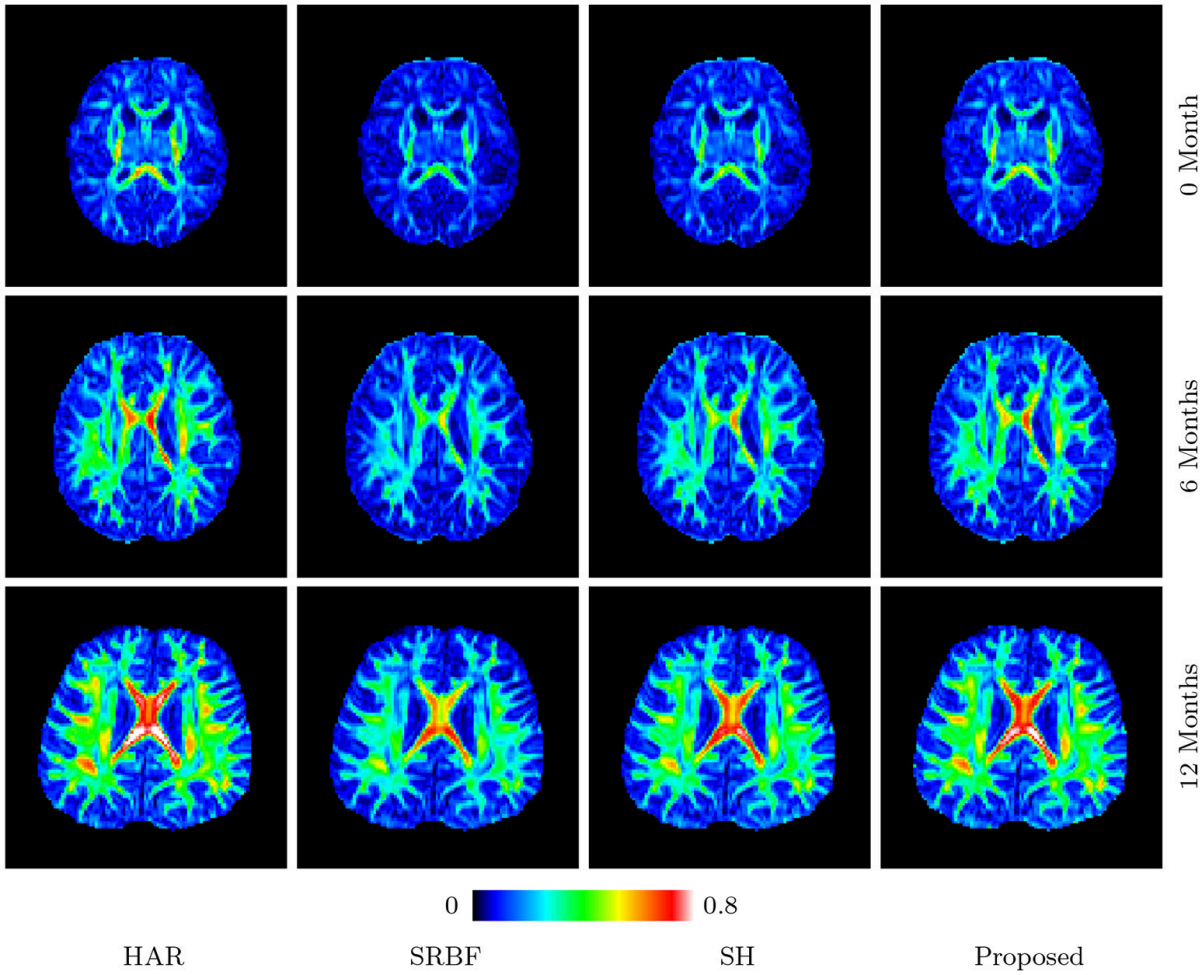
FA Images – Real Data. Comparison of FA images using infant data. The color of FA images represents the FA value, e.g., warmer color means a larger FA value.

**Figure 12:**
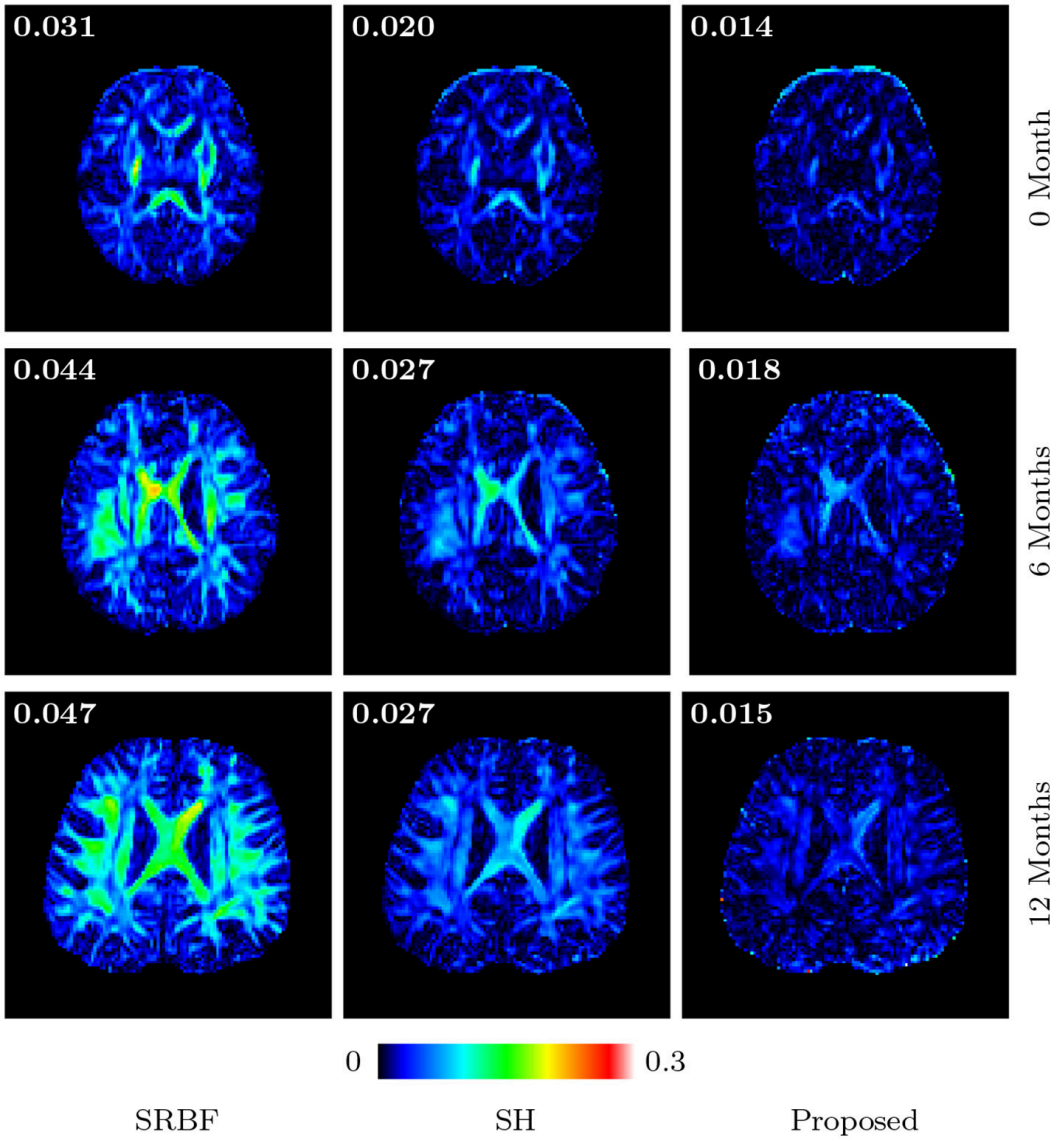
Absolute Difference Maps – Real Data. Comparison of absolute difference maps of FA images using infant data. MAD values are shown at the top left corners.

Figures 11 and 12 further confirm our observation in Figure 5. Our method produces low AD values and gives an FA image that is close to that given by the original HAR data.

#### 3.3.9. Fiber odfs – real data

**Figure 13:**
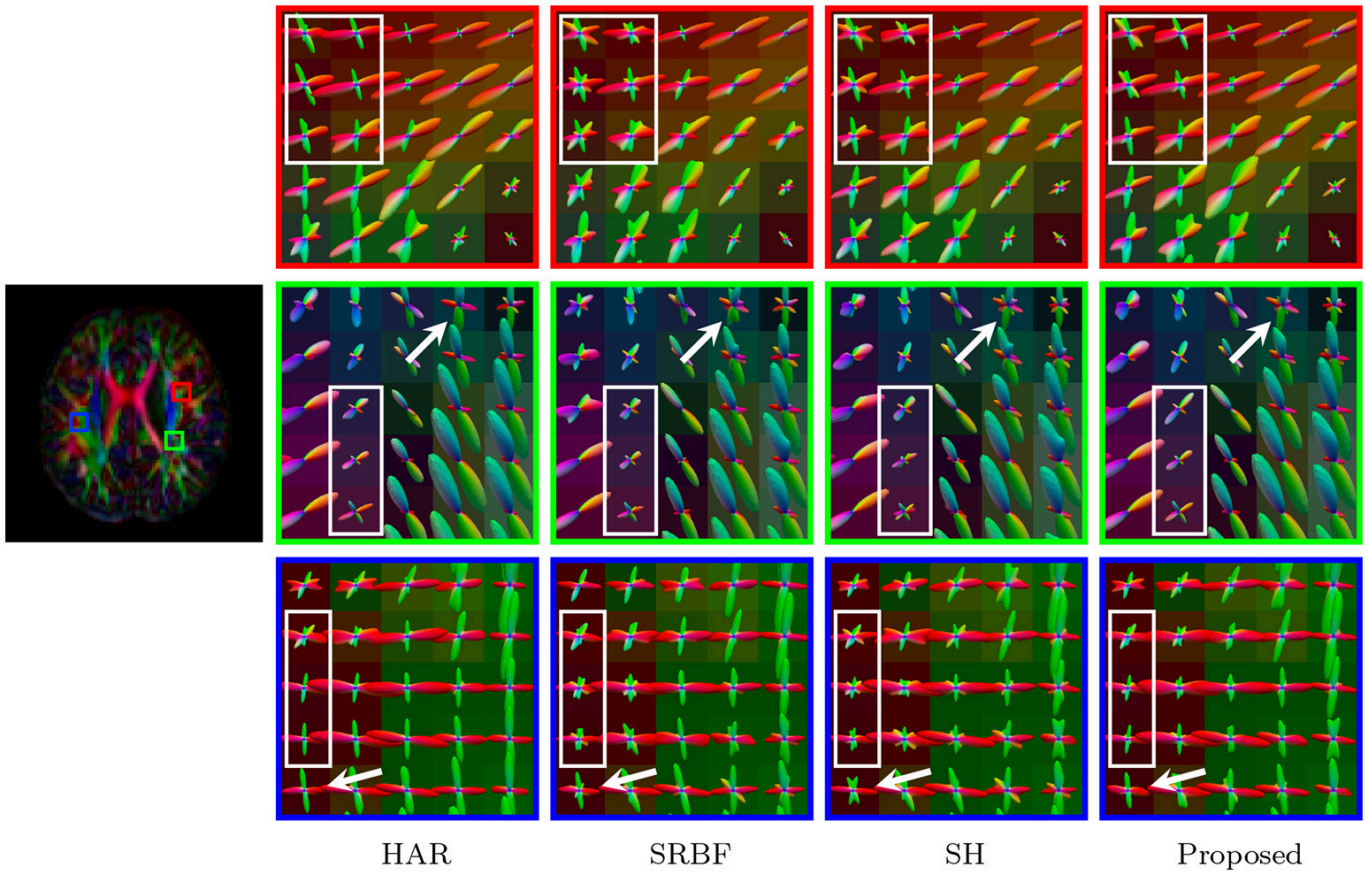
Fiber ODFs – Real Data. Comparison of fiber ODFs using the DMRI data of a 6-Month infant subject.

Figure 13 indicates that our method gives clean and coherent ODFs that are very similar to those given by the original HAR data. In contrast, SRBF interpolation and SH interpolation produce ODFs with a large number of spurious peaks.

## 4. Discussions

Our method is effective because it preserves the sharpness of signal profiles in *q*-space during upsampling. Utilizing non-local smoothness as prior, it takes into account signal similarity in *x*-*q* space and avoids the pitfall of averaging over disparate signals. For the curved white matter structures, considering only signal correlation in *x*-space (i.e., a fixed point in *q*-space) is problematic because the signal changes rapidly across space. On the other hand, considering only signal correlation in *q*-space (i.e., a fixed point in *x*-space) causes smoothing of anisotropic signal profiles due to sharp changes across *q*-space measurements. Our method harnesses the fact that the signal is smooth in the joint *x*-*q* space, even for highly curved structures.

There are some recent works on using compressive sensing (CS) (Baraniuk, 2007) to recover high-resolution (HR) DMRI data from under-sampled *k*-*q* space data. For instance, Mani et al. (2015) performed HR DMRI data reconstruction by imposing sparsity on the coefficients of ODFs and by reducing the total variation (TV) of the HR DMRI data. Cheng et al. (2015) proposed a method, called 6D-CS-DMRI, to recover the ensemble average propagator (EAP) and HR DMRI data simultaneously in a CS framework. The associated ill-posed inverse problem was regularized by the sparsity of the coefficients of EAP, the DW image smoothness enforced by TV, and the sparsity of the wavelet coefficients of DW images. Despite achieving promising performance in recovering HR DMRI data, these methods need dedicated imaging protocols, restricting their widespread application. In contrast, our method enhances the angular resolution post-acquisition and thus avoids special imaging protocols.

In practice, our method is limited by its large memory requirement. The memory issue is mainly caused by the need to store the matching weights used in the fixed-point iteration algorithm. To put this in perspective, if we have *n* *x*-*q* space points, the number of matching weights then becomes *n*×*m*, where *m* is the size of *x*-*q* space search volume. This indicates that the size of matching weights is about *m* times larger than the DMRI data, causing memory issues. A straightforward solution to this problem is to perform angular upsampling in overlapped blocks and then combine the results to form the HAR DMRI data. Another solution is to reduce the number of matching weights to save memory cost. For instance, we can select a certain number of top weights, instead of using all the weights given by neighborhood matching.

## 5. Conclusion

We have presented a regularization framework for *q*-space upsampling. The relationships of signals in *x*-*q* space are used to regularize the inverse problem associated with recovering the HAR DMRI data. Extensive experiments on synthetic and infant DMRI data indicate that our method is able to produce HAR DMRI data with significantly improved quality. Future research effort will be directed to extending the current framework for resolution enhancement in joint *x*-*q* space.

## Author contributions

GC and P-TY implemented the code and designed the experiments. GC drafted the manuscript. P-TY revised the manuscript. WL provided the clinical data. BD, YZ, and DS participated in idea discussion and reviewed the manuscript.

### Conflict of interest statement

The authors declare that the research was conducted in the absence of any commercial or financial relationships that could be construed as a potential conflict of interest.

## Footnotes

^1^http://hardi.epfl.ch/static/events/2013_ISBI/
